# A Novel Statin Compound from Monacolin J Produced Using CYP102A1-Catalyzed Regioselective C-Hydroxylation

**DOI:** 10.3390/ph14100981

**Published:** 2021-09-26

**Authors:** Ngoc Tan Cao, Ngoc Anh Nguyen, Chan Mi Park, Gun Su Cha, Ki Deok Park, Chul-Ho Yun

**Affiliations:** 1 School of Biological Sciences and Biotechnology, Graduate School, Chonnam National University, Yongbong-ro 77, Gwangju 61186, Korea; 187529@jnu.ac.kr; 2 School of Biological Sciences and Technology, Chonnam National University, Yongbong-ro 77, Gwangju 61186, Korea; ngocanh61093@gmail.com (N.A.N.); cmpark0710@gmail.com (C.M.P.); 3 Namhae Garlic Research Institute, 2465-8 Namhaedaero, Gyungnam 52430, Korea; gscha450@gmail.com; 4 Gwangju Center, Korea Basic Science Institute, Gwangju 61186, Korea; kdpark@kbsi.re.kr

**Keywords:** monacolin J, human liver microsomes, statin, HMG-CoA reductase, regioselective hydroxylation

## Abstract

Statins inhibit the 3-hydroxy-3-methyl-glutaryl-coenzyme A reductase (HMG-CoA reductase), which is the rate-limiting enzyme in cholesterol biosynthesis. Statin therapy reduces morbidity and mortality in those who are at high risk of cardiovascular disease. Monacolin J is a statin compound, which is an intermediate in the lovastatin biosynthesis pathway, in the fungus *Aspergillus terreus*. It is also found in red yeast rice, which is made by culturing rice with the yeast *Monascus purpureus*. Monacolin J has a hydroxyl substituent at position C’-8 of monacolin L. Here, a new statin derivative from monacolin J was made through the catalysis of CYP102A1 from *Bacillus megaterium*. A set of CYP102A1 mutants of monacolin J hydroxylation with high catalytic activity was screened. The major hydroxylated product was C-6′a-hydroxymethyl monacolin J, whose structure was confirmed using LC–MS and NMR analysis. The C-6′a-hydroxymethyl monacolin J has never been reported before. It showed a greater ability to inhibit HMG-CoA reductase than the monacolin J substrate itself. Human liver microsomes and human CYP3A4 also showed the ability to catalyze monacolin J in producing the same product of the CYP102A1-catalyzed reaction. This result motivates a new strategy for the development of a lead for the enzymatic and chemical processes to develop statin drug candidates.

## 1. Introduction

Statins are potent inhibitors of the 3-hydroxy-3-methyl-glutaryl-coenzyme A reductase (HMG-CoA reductase), which is the enzyme that catalyzes the rate-limiting step of cholesterol biosynthesis [1,2]. The statins also have other pleiotropic activities, such as anti-inflammatory, antithrombotic, and improved endothelial functions [3]. They reduce cardiovascular disease and mortality in people at high risk of cardiovascular disease [4,5]. Studies using statins for the treatment of hyperlipidemia demonstrated their efficacy in lowering triglycerides and low-density lipoprotein cholesterol. Recently, their use in cancer therapy was noticed as a tumor suppressor or adjuvant to chemotherapy. Several studies suggested that statins may reduce cancer mortality and may demonstrate some protective effects through the use of chemotherapy and radiation-induced cardiovascular toxicity [6]. Today, the search for new substances derived from statins to support treatment and to create more diverse products is essential in research [7,8].

Currently, seven statins are available in major international markets: lovastatin, simvastatin, pravastatin, fluvastatin, atorvastatin, rosuvastatin, and pitavastatin [9]. The statins share similar chemical characteristics but differ in their molecular synthesis, solubility, and pharmacokinetic behavior. Among them, lovastatin (also known as monacolin K) is the only natural statin that is mainly produced by *Aspergillus terreus* strains. The biosynthetic pathway for lovastatin from *A. terreus* includes the three key intermediates of dihydromonacolin L, monacolin L, and monacolin J [10]. The 2-methylbutyryl side chain of lovastatin is synthesized by a polyketide synthase, namely, LovF, and transferred to the C-8′ hydroxyl group of monacolin J by LovD, which is an acyltransferase. Inactivation of either LovD or LovF leads to the accumulation of the monacolin J. Pravastatin is derived from compactin, which is a natural analog of lovastatin that is produced by *Penicillium citrinum*, via biotransformation. Simvastatin can be efficiently synthesized from monacolin J (a lovastatin analog without the side chain at C-8′ hydroxyl group) via a process that uses the *A. terreus* acyltransferase LovD [11].

On the other hand, monacolin J itself is a natural statin compound that is found in red yeast rice. It is made by culturing rice with various strains of the yeast *Monascus purpureus* [12]. It has the ability to inhibit cholesterol biosynthesis [13]. Monacolin J is a precursor of lovastatin, which is an important drug for treating hypercholesterolemia [14] and has potential neuroprotective activities [15].

Interestingly, monacolin J was used as an intermediate to produce six novel statin derivatives by attaching different side chains at the C-8′ hydroxyl residue [15]. These novel derivatives were screened for their hypocholesterolemic and neuroprotective activities. One derivative showed a very low HMG-CoA reductase *IC*_50_ and a high protection rate for oxidative-stress-induced neuron cell death. This result showed that monacolin J can be used as a valuable intermediate for the synthesis of novel drug candidates via biotechnological and chemical procedures.

CYP102A1 (P450 BM3) from *Bacillus megaterium* has attracted considerable interest due to its excellent enzymatic properties, such as higher catalytic activity, more broad substrate scope, and higher catalytic efficiency compared to other P450s. With a large number of crystal structures, the soluble expression in *Escherichia coli* and its self-sufficient characteristics make CYP102A1 a P450 prototype for studying the structure–function relationships of P450s. It has the ability to participate in catalyzing relatively diverse reactions [16,17,18]. In particular, CYP102A1 is a biocatalyst that catalyzes the production of human metabolites [19], which are useful in chemical biosynthesis and pharmaceutical fields. CYP102A1 catalyzes the 6′β-hydroxylation of lovastatin and simvastatin to make human metabolites from them [19]. Multistep chemical synthesis of drugs, drug metabolites, and fine chemicals is laborious, cost expensive, and environmentally unfriendly.

**Figure 1:**
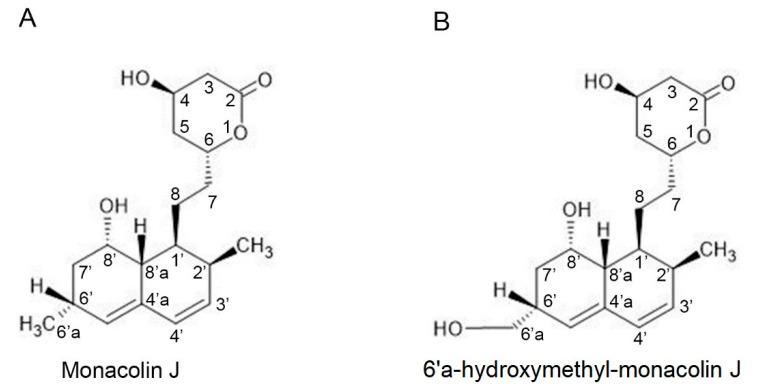
Chemical structures of monacolin J and 6′a-hydroxymethyl monacolin J. (**A**) Chemical structure of monacolin J. (**B**) Chemical structure of a major product from monacolin J that was catalyzed using CYP102A1 in the presence of NADPH. The structure of the product was found to have an OH group attached to the C-6′a position, while the original monacolin J structure had an H_3_ group.

Using cytochrome P450 (CYP or P450) enzymes, this study was conducted to synthesize new compounds from monacolin J through greener biological pathways. Here, we found that the CYP102A1 mutants could catalyze regioselective hydroxylation of monacolin J. More specifically, the hydroxylated product was characterized as a new compound with the OH group attached at position C-6′a of monacolin J, which has never been reported before (Figure 1). The potential of the new hydroxylated product was evaluated by means of an HMG-CoA reductase inhibition assay by comparing it with monacolin J. The result showed that 6′a-hydroxymethyl monacolin J has the ability to inhibit HMG-CoA reductase at higher levels than monacolin J. This result suggested a new research approach to make a lead to produce new derivatives of statin drugs.

## 2. Results

### 2.1. Monacolin J Oxidation Using CYP102A1 and Identification of the Major Product

**Figure 2:**
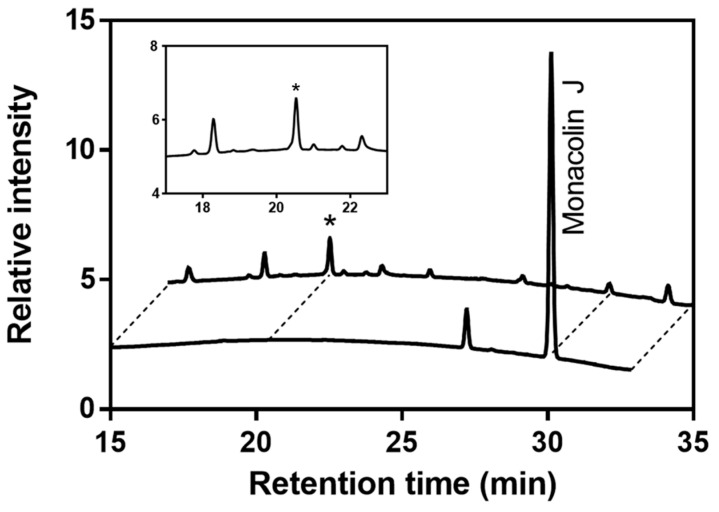
HPLC chromatograms of monacolin J and its products when using CYP102A1 M697. The peaks of the reaction mixtures were identified by comparing their retention times: a major product of monacolin J (*t*_R_ = 20.4 min) and monacolin J (*t*_R_ = 30.2 min). The star marks the major product.

**Figure 3:**
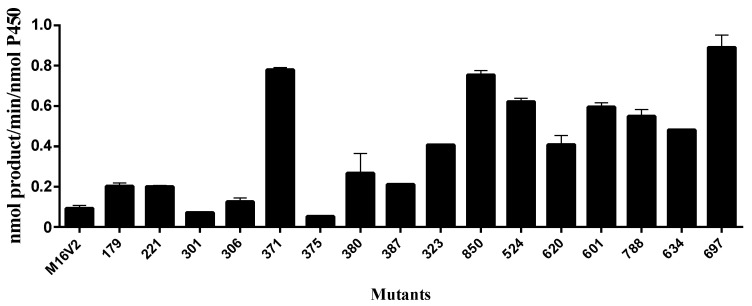
Hydroxylation rate of the monacolin J catalyzed using CYP102A1 mutants. The M697 showed the highest hydroxylation activity among the tested mutants. The rates of the major product formation using 17 mutants are presented as the mean ± SEM of triplicate measurements.

To determine the ability of CYP102A1 to hydroxylate monacolin J, a set of its mutants were reacted with monacolin J (200 μM) at 37 °C. The 60 mutants that were used to find highly active mutants toward monacolin J were selected based on our previous works showing their catalytic activities on several natural substrates [20,21,22,23]. Each mutant had amino acid substitutions relative to wild-type (WT) CYP102A1, as shown in Supplementary Table S1. The effect of CYP102A1 mutants on monacolin J was checked using HPLC (Figure 2). Seventeen of the 60 mutants tested here were shown to have hydroxylation activity (>0.05 min^−1^) toward monacolin J (Figure 3), with M697 exhibiting the highest hydroxylation activity among the tested mutants (Figure 2). One major metabolite was observed at a retention time of 20.4 min. The other mutants did not provide any apparent catalytic activity (<0.01 min^−1^). Optimal pH for production of the hydroxylated product was 7 (Figure S1).

**Figure 4:**
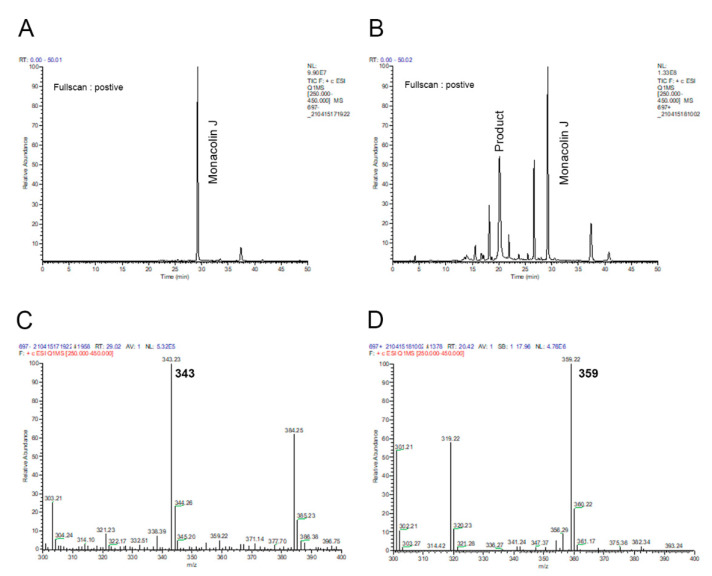
LC–MS results of the product of monacolin J when using CYP102A1. LC–MS chromatogram of monacolin J (**A**) and its reaction mixture catalyzed using CYP102A1 in the presence of NADPH (**B**). The MS spectra demonstrated that the *m*/*z* values of the protonated molecular ions of monacolin J (**C**) and its hydroxylated product (**D**) were 343 and 359, respectively.

To characterize the chemical structure of the major product, the reaction was analyzed using LC–MS (Figure 4). A full-scan chromatogram of the reaction mixture of monacolin J with M697 showed that monacolin J (Figure 4A) was converted to several products (Figure 4B). The results of the LC–MS analysis indicated that the major product (*m/z* = 359) (*t*_R_ = 20.42 min) was hydroxylated (Figure 4D) when compared to the substrate, namely, monacolin J (*m/z* = 343) (Figure 4C).

**Figure 5:**
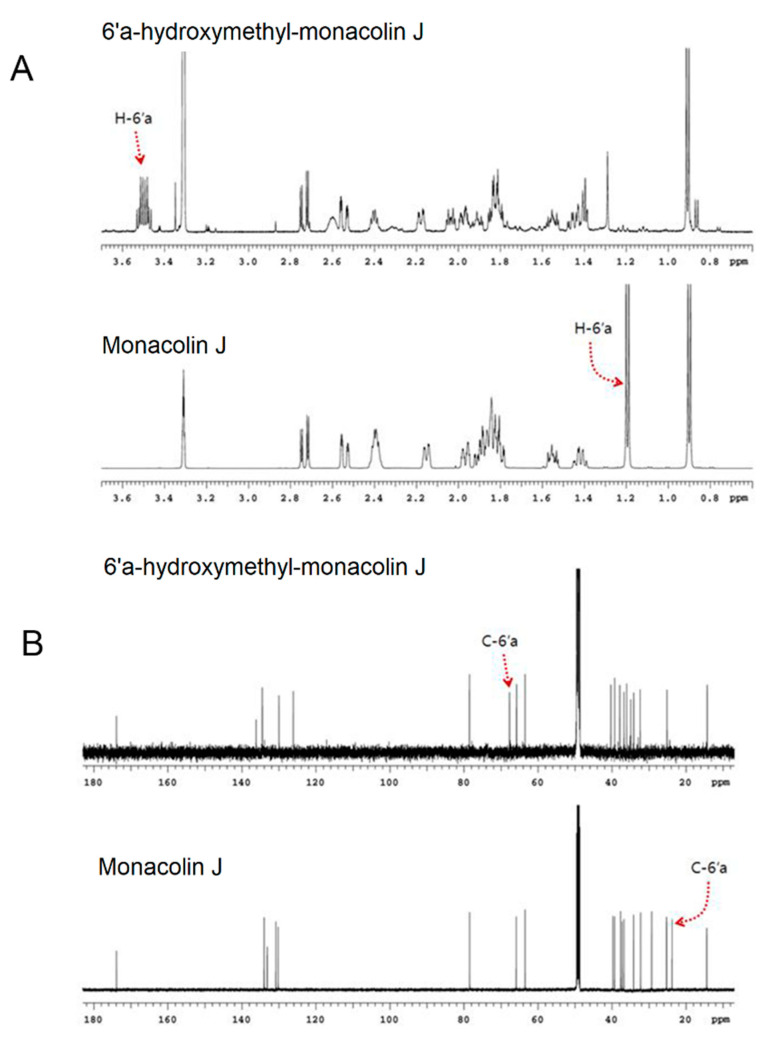
NMR results of the major product of monacolin J when using CYP102A1. (**A**) Expanded ^1^H NMR spectra of monacolin J and its product, namely, 6′a-hydroxymethyl monacolin J (0.7~3.7 ppm region). Chemical shift of methyl protons at C-6′a of monacolin J moved downfield at 3.50 ppm. (**B**) ^13^C NMR spectra of monacolin J and its product, namely, 6′a-hydroxymethyl monacolin J.

After the major product was collected and dried, its spectral assignments were carried out with ^1^H NMR, ^13^C NMR, and two-dimensional experiments. Figure 5 shows the one-dimensional ^1^H and ^13^C NMR spectra of monacolin J and its major product. The ^1^H NMR results of monacolin J that were obtained in CD_3_OD at 600 MHz were in good agreement with the previous assignment of monacolin J [13]. The chemical structure of the product was identified as 6′a-hydroxymethyl monacolin J. In the ^1^H and ^13^C NMR spectra of 6′a-hydroxymethyl monacolin J, methyl protons (1.19 ppm, doublet) and carbon (23.78 ppm) at the C-6′a location of the original substrate, namely, monacolin J, disappeared and new CH_2_ peaks (^1^H: 3.50 ppm, ^13^C: 67.71 ppm) appeared (Figure 5). Detailed ^1^H and ^13^C assignments according to the analysis of two-dimensional spectra (COSY, HSQC, and HMBC) are listed in Tables S2 and S3.

### 2.2. Kinetics Parameters and Total Turnover Numbers (TTNs) of Monacolin J Hydroxylation Using CYP102A1

Four mutants (M16V2, M380, M371, and M697) with high monacolin J hydroxylation activity among the tested CYP102A1 mutants were used to determine the kinetic parameters. Table 1 shows the steady-state kinetics of 6′a-hydroxymethyl monacolin J formation. M697 showed the highest *k*_cat_ value of 1.01 min^−1^ and the lowest *K*_m_ value of 92 μM among the tested CYP102A1 mutants. M697 also showed the highest catalytic efficiency when converting monacolin J to 6′a-hydroxymethyl monacolin J.

**Figure 6:**
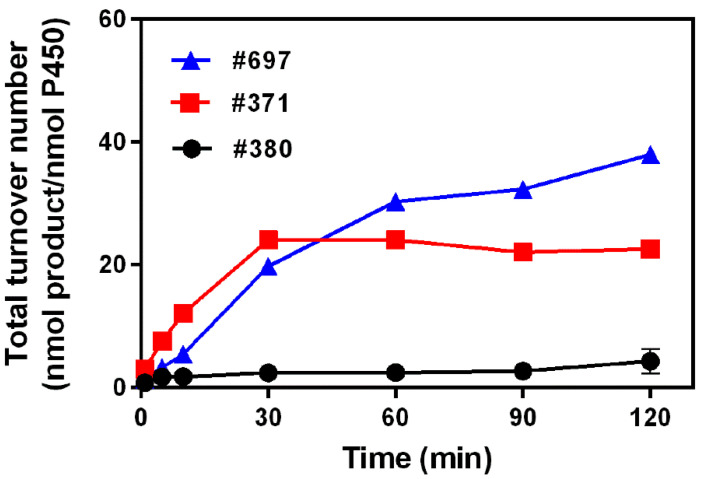
Time profiles of 6′a-hydroxymethyl-monacolin J formation, which was catalyzed using M380, M371, and M697. TTN values were determined using HPLC after accomplishing the reactions at indicated times at 37 °C. The formation of 6′a-hydroxymethyl monacolin J was measured in the reaction mixtures containing 0.2 μM of P450, an NADPH regeneration system, and monacolin J (0.30 mM) for a total volume of 0.25 mL.

When the time profiles of the reactions with three mutants were determined by prolonging the incubation time from 1 to 120 min, the formation of 6′a-hydroxymethyl monacolin J increased with the reaction time. The total turnover number (TTN, nmol product/nmol enzyme) of M697 reached the highest levels of 38 at 120 min of reaction time. For M371, it showed the maximum TTN value at 30 min of 22. Meanwhile, M380 showed the lowest values compared with M371 and M697 (Figure 6).

### 2.3. Hydroxylation of Monacolin J Catalyzed Using Human Liver Microsomes

**Figure 7:**
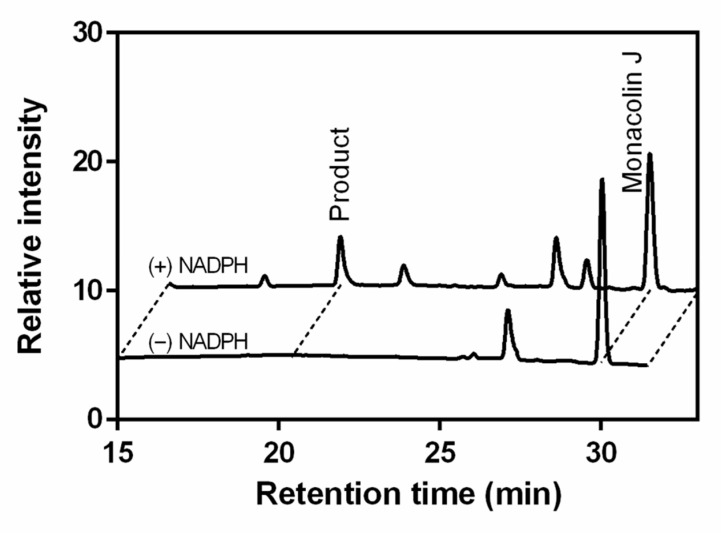
HPLC chromatograms of monacolin J and its products when using HLMs. The reaction mixtures contained 0.4 μM HLMs and 0.2 mM monacolin J with or without an NADPH regenerating system. The major product and monacolin J appeared at 20.4 and 30.2 min, respectively.

**Figure 8:**
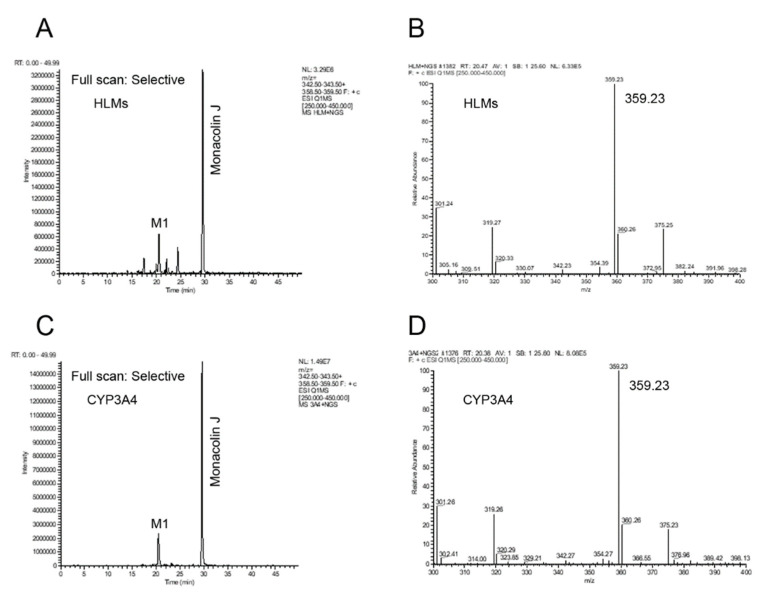
LC–MS results of the metabolite of monacolin J when using HLMs and CYP3A4. LC–MS chromatogram of monacolin J that was catalyzed using HLMs (**A**) and CYP3A4 (**C**) in the presence of NADPH. The MS spectra demonstrated that the *m*/*z* value of the protonated molecular ion of the major product from both of HLMs (**B**) and CYP3A4 (**D**) was 359, which is the same as that produced with M697.

Monacolin J is found in red yeast rice [13]. Therefore, during the consumption of red rice, the oxidative metabolism of monacolin J with the enzymes occurs in the human liver. In the human liver microsomes (HLMs), there are some prominent P450 enzymes that are involved in oxidation processes, such as the CYPs 1A2, 3A4, 2C19, and 2D6 [24,25]. This study of the ability of P450 enzymes in HLMs to catalyze monacolin J hydroxylation adds to the knowledge about the monacolin J oxidative metabolism in the body. When monacolin J was incubated with HLMs and recombinant human CYPs, such as the CYPs 2C9, 2E1, 2C19, 3A4, and 2D6, only HLMs and CYP3A4 (0.040 nmol product min^−1^ nmol P450^−1^) showed the apparent catalytic activity of monacolin J hydroxylation (Figure 7 and Figure 8). The CYPs 2C9, 2E1, 2C19, and 2D6 did not show catalytic activity (<0.005 min^−1^). The major product peak showed the same retention time (*t*_R_ = 20.4 min) as that produced by the CYP102A1 M697 mutant. When the kinetic parameters of HLMs for monacolin J hydroxylation were determined, the *k*_cat_ and *K*_m_ values were 0.10 min^−1^ and 424 μM, respectively. The monacolin J hydroxylation reactions that were catalyzed by HLMs and CYP3A4 were also examined using LC–MS analysis (Figure 8) and similar results to those of the CYP102A1 M697 were obtained (Figure 4). The major product (*m/z* = 359) was a hydroxylated product when compared to the substrate, namely, monacolin J (*m/z* = 343). This result indicated that HLMs had the same regioselective hydroxylation activity toward monacolin J to produce the same hydroxylated product with M697. Taken together, it is reasonable to consider that the human P450 enzymes can participate in the catalytic hydroxylation of monacolin J in the HLMs.

### 2.4. Inhibitory Effects of Monacolin J and Its Product on HMG-CoA Reductase

**Figure 9:**
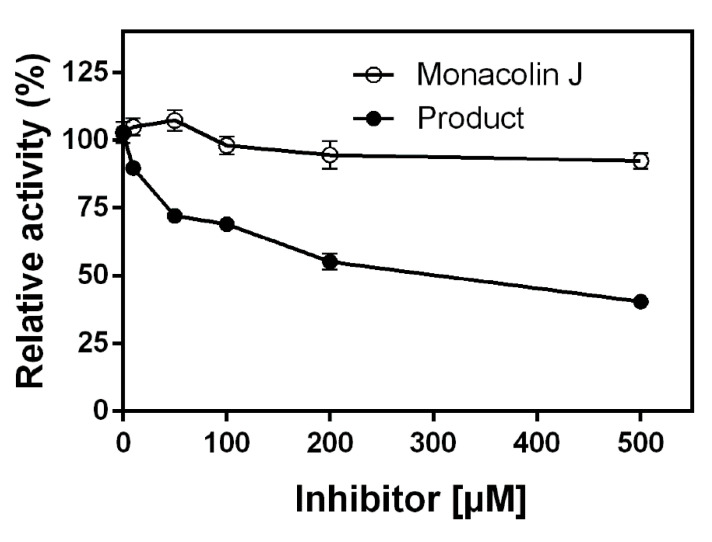
Inhibitory effects of monacolin J and its product on HMG-CoA reductase. The inhibitory activity of monacolin J and its product (6′a-hydroxymethyl monacolin J) on HMG-CoA reductase was determined. Monacolin J and its product were used at concentrations up to 500 μM. Data are shown as the means (*n* = 3) ± SEM.

The inhibitory effect of monacolin J and its product, namely, 6′a-hydroxymethyl monacolin J, on HMG-CoA reductase was investigated. Monacolin J and its product inhibited the activity of HMG-CoA reductase as their concentrations increased in the range of 5–500 μM (Figure 9). The results showed that the product inhibited HMG-CoA reductase activity more strongly than the monacolin J substrate. The highest inhibitions of monacolin J and 6′a-hydroxymethyl monacolin J were 9.7 and 60.7%, respectively, at a concentration of 500 μM. The *IC*_50_ of 6′-hydroxymethyl monacolin J on HMG-CoA reductase activity was 285 μM. The product, namely, 6′a-hydroxymethyl monacolin J, was found to show great potential when compared to the substrate, namely, monacolin J, which did not show apparent inhibitory effects up to 500 μM (<10%). The hydroxylated monacolin J can become a new substance with high inhibitory activity against HMG-CoA reductase, and it can be a good candidate for statin drugs in future research.

## 3. Discussion

Cardiovascular diseases (CVDs) are the leading cause of death around the world, taking an estimated 17.9 million lives in 2019, representing 32% of all global deaths according to the World Health Organization [26]. CVDs are a group of disorders of the heart and blood vessels, including coronary heart disease and cerebrovascular disease. More than four out of five CVD deaths are due to heart attacks and strokes. High levels of serum cholesterol, specifically low-density lipoprotein cholesterol (LDL-C), are associated with an increased risk of atherosclerotic CVDs. LDL-C reduction with statins has been a popular therapy for CVD prevention because statin treatment leads to reduced serum levels of total cholesterol, LDL-C, and triacylglycerols [27,28]. The significance of statins was recognized in the primary and secondary prevention of atherosclerotic CVDs [28,29,30,31,32,33]. In primary prevention, statins substantially decrease CVD morbidity, but only moderately reduce CVD mortality [27,28,29,30,31,32,33]. Statin use for primary prevention can be effective for individuals at high risk of CVD [28]. The beneficial effects of statins in secondary prevention were reported to reduce all-cause, CVD mortality, and cardiovascular events [29,31,32,33].

In this study, we made a novel hydroxylated product of monacolin J that was catalyzed using CYP102A1. Based on the results obtained and analyzed using HPLC, LC–MS, and NMR methods, we confirmed that the hydroxylated product of monacolin J was a completely new substance that has never been reported before. The position of the OH group of the metabolite was attached to the C-6′a position (Figure 1).

Although the chemical, pharmacokinetic, and pharmacodynamic properties of several popular statin compounds (i.e., atorvastatin, cervastatin, fluvastatin, lovastatin, simvastatin, pravastatin, rosuvastatin, and pitavastatin) are well known [34], the properties of monacolin J have not been studied extensively. The inhibitory effects of monacolin J and 6′a-hydroxymethyl monacolin J on HMG-CoA reductase seem to be weaker than those of other statins (such as simvastatin, lovastatin, fluvastatin, and atorvastatin) and their hydroxylated products [21]. It was reported that the *IC*_50_ of the acid form (open hydroxycarboxylate) of monacolin J on HMG-CoA reductase activity was 3.3 μM [13]. After assessing the effect of monacolin J (lactone form) on HMG-CoA reductase in this study, we could not obtain the *IC*_50_ value of monacolin J because only 9.7% of the HMG-CoA reductase activity was inhibited at a concentration of 500 μM (Figure 9). This result suggested that the acid form of monacolin J is an active form in vivo. The *IC*_50_ of 6′-hydroxymethyl monacolin J on HMG-CoA reductase activity was 285 μM. The product, namely, 6′a-hydroxymethyl monacolin J, was found to show great potential when compared to the substrate, namely, monacolin J, which did not show apparent inhibitory effects up to 500 μM (<10%).

The *IC*_50_ values of statins on HMG-CoA reductase activity depend on the report and were in the range from several nanomolar to several micromolar levels. It was reported that rosuvastatin’s ability to inhibit 50% of HMG-CoA reductase activity occurs at the lowest concentration (*IC*_50_ = 5.4 nM) among the tested statins, followed by atorvastatin (8.2 nM) [35]. Other statins showed higher *IC*_50_ values: simvastatin (11.2 nM), fluvastatin (27.6 nM), and pravastatin (44.1 nM). Another report showed the *IC*_50_ of lovastatin was 390 μM [36]. Other studies reported that the *IC*_50_ of atorvastatin and fluvastatin are between 40 and 100 nM and for pravastatin, simvastatin, and lovastatin, the *IC*_50_ is between 100 and 300 nM [37]. Previously, we found that the statins and their metabolites show different inhibitory effects on HMG-CoA reductase [38]. The *IC*_50_ values of other statins are as follows: 6β-OH simvastatin (2.0 μM), lovastatin (0.95 μM), 6β-OH lovastatin (1.2 μM), fluvastatin (0.38 μM), atorvastatin (56 nM), and 4-OH atorvastatin (1.2 μM).

Although all statins share a common mechanism of action, they differ in terms of their chemical structures, pharmacokinetic and pharmacodynamic properties, and bioavailabilities [34]. Their chemical structures influence the metabolism processes of their absorption, distribution, metabolism, and excretion. Lovastatin, pravastatin, and simvastatin have elimination half-lives of 1–3 h. Atorvastatin, fluvastatin, pitavastatin, and rosuvastatin show elimination half-lives ranging from 1 h for fluvastatin to 19 h for rosuvastatin. The bioavailability of the statins differs greatly, from 5% for lovastatin and simvastatin to 60% for pitavastatin. Clinical studies have demonstrated rosuvastatin to be the most effective for reducing low-density lipoprotein cholesterol, followed by atorvastatin, simvastatin, and pravastatin.

The metabolism processes of the statins in the human body should be considered regarding their functions as they depend on the chemical structure of each statin [39]. Human P450s have an important role in the clinical drug metabolism of statin drugs [40,41]. CYP3A4 is responsible for atorvastatin, simvastatin, and lovastatin metabolism. CYP2C9 metabolizes fluvastatin and, to a lesser degree, pitavastatin. Rosuvastatin, pravastatin, and pitavastatin undergo negligible metabolic interactions with P450 enzymes. In addition, we should consider the issue of active metabolites of statins as several statins are administered as prodrugs and converted to active metabolites in the liver [9,42].

The results obtained in this study showed that HLMs and CYP3A4 participate in the oxidation of monacolin J. The major product of monacolin J oxidation using HLMs and CYP3A4 was the same as that of bacterial CYP102A1. CYP3A4 is one of the most important P450 isozymes in the metabolism of statin drugs [43]. CYP3A4 is abundant in the liver and small intestine and can inhibit or activate drug properties during treatment. This result suggests that the 6′a-hydroxymethyl monacolin J is produced by HLMs and CYP3A4 in the liver when red rice was consumed and that it may provide health benefits by reducing cholesterol biosynthesis.

Even though the inhibitory effect of 6′a-hydroxymethyl monacolin J on HMG-CoA reductase is not comparable to previously known statins, this new compound can be used to develop new statin drug candidates. Previously, the C-8′ hydroxyl residue of monacolin J was used as a target site to produce novel statin derivatives by attaching different side chains [15]. One derivative had a very low HMG-CoA reductase *IC*_50_ and a high protection rate for oxidative-stress-induced neuron cell death. The new statin compound, namely, 6′a-hydroxymethyl monacolin J, can be used as a lead to make several new statin derivatives by using enzymatic (i.e., acyltransferase) and chemical modifications. Taken together, 6′a-hydroxymethyl monacolin J can be considered as a valuable intermediate for the synthesis of novel drug candidates using biotechnological and chemical procedures.

To the best of our knowledge, this is the first report to identify 6′a-hydroxymethyl monacolin J, which is a major product of monacolin J that is catalyzed using CYP102A1. The kinetic studies suggest that CYP102A1 M697 had the highest turnover for monacolin J among mutants (Table 1). With a new structure, 6′a-hydroxymethyl monacolin J can be considered an important compound for further studies on its influence on the ability to treat or the ability to participate in other reactions in the body. The experimental data suggested that 6′-hydroxymethyl monacolin J was a stronger inhibitor of HMG-CoA reductase when compared with monacolin J, although it had a much higher *IC*_50_ value than other statins and their metabolites. This study suggests a possibility that the product, namely, 6′a –hydroxymethyl monacolin J, can be used as a lead for the production of new statin derivatives. Further modification of 6′a-hydroxymethyl monacolin J using enzymatic and chemical methods should be applicable in producing diverse statin derivatives.

## 4. Materials and Methods

### 4.1. Materials

Monacolin J was purchased from 4Chem Laboratory (Gyeonggi-do, Korea); in addition, β-nicotinamide adenine dinucleotide phosphate (NADP^+^), glucose-6-phosphate, glucose-6-phosphate dehydrogenase, acetonitrile, methanol, and ethyl acetate were purchased from Sigma-Aldrich (St. Louis, MO, USA). Other chemicals used in this study were of the highest grade commercially available and used without further purification.

HLMs were purchased from ThermoFisher Scientific (Waltham, MA, USA) and antibodies against CYP3A4 and 2C19 were prepared in the previous work [44].

The recombinant human P450s were heterologously expressed in *E. coli* with a pCW vector containing human P450 cDNA and rat NADPH-P450 reductase (CPR). The pCW vectors expressing both P450 and CPR were constructed in previous studies: CYPs 1A2, 1B1, 2E1, 3A4, 2C19, 2D6, and 2A6 [45,46]. Membrane fractions expressing each human P450 and rat CPR were prepared as described previously [45,46] and used for the catalytic activity of monacolin J hydroxylation.

### 4.2. Hydroxylation of Monacolin J Catalyzed Using CYP102A1

A set of CYP102A1 was used to screen hydroxylation activities toward monacolin J. The CYP102A1 variants used in this study were chosen based on previous studies. The mutants A5-M697 have mutations at the heme domains of M16V2 and contain the underlined 20 mutated amino acids of the V2 reductase domain of M16V2 (Table S1). M16V2 is a chimeric variant of the M16 heme domain and a reductase domain of the V2 variant [47].

The hydroxylation reactions of monacolin J that were catalyzed using CYP102A1 and human P450/rat CPR containing membrane fractions were done as follows: The reaction contained 0.2 nmol P450 enzyme, an NADPH regeneration system (NGS, final concentration: 10 mM glucose-6-phosphate, 0.50 mM NADP^+^, and 1.0 IU yeast glucose-6-phosphate dehydrogenase per mL), and monacolin J (0.3 mM) in a final volume of 0.25 mL of a potassium phosphate buffer (0.1 M, pH 7.4). The reaction mixtures were incubated at 37 °C for 30 min and stopped by adding 0.6 mL of cold ethyl acetate, followed by centrifugation (1000× *g*, 20 min) to separate the organic and aqueous layers [48,49]. The organic layer was concentrated under a stream of nitrogen, and the residue was dissolved in 180 µL of mobile phase A:B (8:2, *v*/*v*). Mobile phase A contained distilled water and 0.1% formic acid, mobile phase B was 100% acetonitrile, and 20 µL of samples were injected onto a Gemini C18 column (4.6 mm × 150 nm, i.d. 5 µm; Phenomenex, Torrance, CA, USA). The flow rate of the mobile phase was 0.7 mL/min, which was achieved via a gradient pump (LC-20AD; Shimadzu, Kyoto, Japan) using an increasing stepwise gradient of acetonitrile (mobile phase B) from 15 to 50% (*v*/*v*) over 55 min: 0–5 min, 15% acetonitrile; 5–25 min, 50% acetonitrile; 25–45 min, 50% acetonitrile, 45–50 min, 15% acetonitrile; and 50–55 min, 15% acetonitrile. The detection was performed at 238 nm.

For the kinetic analysis of monacolin J hydroxylation using mutants, the reaction contained a 0.2 nmol CYP enzyme, an NADPH regeneration system, and monacolin J (0.05−0.5 mM) in a final volume of 0.25 mL of a potassium phosphate buffer (0.1 M, pH 7.4). The reaction mixtures were incubated at 37 °C for 30 min. The kinetic parameters *K*_m_ and *k*_cat_ were calculated through nonlinear regression analysis with GraphPad Prism software (GraphPad, Software Inc., San Diego, CA, USA). The equation for Michaelis–Menten kinetics was applied.

To study the TTNs for the concentration, 500 µM of monacolin J was used. The reaction was started by adding an NADPH-generating system and incubating at 1, 5, 10, 20, 30, 60, 90, 120, and 150 min at 37 °C. The formation rate of the hydroxylated product of monacolin J was determined using HPLC, as described above.

Calibration standards of monacolin J and lovastatin were constructed from a blank sample (a reaction mixture without a substrate and internal standard), nine samples of monacolin J, and nine samples of lovastatin with concentrations of 0.2–500 μM (Figure S2). The peak area ratio of monacolin J to the internal standard (lovastatin) was linear with respect to the analyte concentration over the range of 0.2–500 μM. Quantitation of the product was done by comparing the peak areas of the major product to the mean peak areas of the internal standard (50 μM). The detection limit of the product was 5 μM.

### 4.3. LC–Mass Spectrometric Analysis of a Product of Monacolin J

To characterize the monacolin J products using CYP102A1, LC–MS analysis of the metabolites was executed for the comparison of fragmentation patterns and LC profiles with authentic compounds. M697 was incubated with 200 µM of monacolin J at 37 °C for 1 h with an NADPH-generating system, and injection samples were prepared as described above. An aliquot (7 µL) of this solution was injected into the LC column. LC–MS analysis was carried out in electrospray ionization (negative) mode on a Shimadzu LCMS-2010 EV system (Shimadzu Corporation, Japan) with LCMS solution software. The separation was performed on a Shim-pack VP-ODS column (2.0 mm × 250 mm, Shimadzu Corporation, Japan). Mobile phase A was water containing 0.1% formic acid, mobile phase B was acetonitrile, and mobile phase A/B run with a gradient, as described in the previous section, was delivered at a flow rate of 0.7 mL/min. The interface and detector voltages were 4.4 and 1.7 kV, respectively.

### 4.4. NMR Spectroscopy Analysis of Monacolin J Product

NMR experiments were performed at 25 °C on a Varian VNMRS 600 MHz NMR spectrometer equipped with a carbon-enhanced cryogenic probe. Methanol-d_4_ was used as the solvent, and chemical shifts for proton and carbon-13 NMR spectra were measured in parts per million (ppm) relative to tetramethylsilane (TMS). All of the NMR experiments were carried out with standard pulse sequences in the VNMRJ (v. 3.2) library and processed with the same software.

### 4.5. Inhibitory Effects of Monacolin J and Its Metabolite on HMG-CoA Reductase

The experiment was conducted on an HMG-CoA reductase assay kit from Sigma-Aldrich. Monacolin J and its product were used as an inhibitor with concentrations ranging from 5–1000 μM. Reactions were conducted and measured using a spectrophotometer at 340 nm.

## 5. Conclusions

In this study, the conversion of monacolin J to 6′a-hydroxymethyl monacolin J was investigated based on the catalysis of bacterial CYP102A1, as well as HLMs and human CYP3A4. Monacolin J is a statin compound and a precursor of lovastatin biosynthesis in the fungi *Aspergillus terreus*. It is also found in red yeast rice, which is made by culturing rice with the yeast *M. purpureus*. Here, new statin compounds from monacolin J were made through the catalysis of CYP102A1 from *B. megaterium*. The catalytic ability of CYP102A1 was found to catalyze regioselective hydroxylation reaction toward monacolin J. The product, namely, C-6′a-hydroxymethyl monacolin J, demonstrated the ability to inhibit HMG-CoA reductase better than the monacolin J substrate itself. HLMs and CYP3A4 also showed the ability to catalyze monacolin J by producing the same product of the CYP102A1-catalyzed reaction. Based on the achieved results, we can use this strategy to produce a lead compound to create new statin derivatives with high potentials for therapeutic abilities.

## Figures and Tables

**Table 1:** Kinetic parameters of monacolin J hydroxylation using M380, M371, M697, and CYP16V2.

Enzymes	*k_cat_* (min^−1^)	*K_m_* (μM)	*k_cat_/K_m_* (min^−1^ μM^−1^)
M16V2	0.086 ± 0.025	117 ± 97	0.00074 ± 0.00065
M380	0.24 ± 0.07	656 ± 285	0.00037 ± 0.00019
M371	0.88 ± 0.26	527 ± 256	0.0017 ± 0.0009
M697	1.01 ± 0.14	92 ± 42	0.011 ± 0.005

## Data Availability

Data are contained within the article and Supplementary Materials.

## Supplementary Materials

The following are available online at https://www.mdpi.com/article/10.3390/ph14100981/s1. Figure S1; pH dependence of monacolin J formation using CYP102A1, Figure S2: Standard curves of the internal standard lovastatin and monacolin J, Table S1: The amino acid sequence of M16V2 and CYP102A1 mutants, Table S2: Detailed ^1^H and ^13^C assignments according to the analysis of two-dimensional spectra (COSY, HSQC, and HMBC) of monacolin J, Table S3: Detailed ^1^H and ^13^C assignments according to the analysis of 2-dimensional spectra (COSY, HSQC, and HMBC) of the major product.
