# Supplementary material for: A Novel Statin Compound from Monacolin J Produced Using CYP102A1-Catalyzed Regioselective C-Hydroxylation
Source: Pharmaceuticals (Basel). 2021 Sep 26;14(10):981. doi: 10.3390/ph14100981 (PMC8541633; doi:10.3390/ph14100981)
Supplement: Supplementary file 1 [file pharmaceuticals-14-00981-s001.zip › pharmaceuticals-1336667-supplementary.pdf]

pharmaceuticals

Supporting Information

A Novel Statin Compound from Monacolin J Produced Using CYP102A1-Catalyzed Regioselective C-Hydroxylation

Ngoc Tan Cao [1], Ngoc Anh Nguyen [2], Chan Mi Park[2], Gun Su Cha [3], Ki Deok Park[4], and Chul-Ho Yun [1,2]*

[1] School of Biological Sciences and Biotechnology, Graduate School, Chonnam National University, Yongbong-ro 77, Gwangju 61186, Republic of Korea; 187529@jnu.ac.kr (N.T.C.).
[2] School of Biological Sciences and Technology, Chonnam National University, Yongbong-ro 77, Gwangju 61186, Republic of Korea; ngocanh61093@gmail.com (N.A.N.); cmpark0710@gmail.com (C.M.P.).
[3] Namhae Garlic Research Institute, 2465-8 Namhaedaero, Gyeongsangnamdo 52430, Republic of Korea; gscha450@gmail.com (G. S.C.).
[4] Gwangju Center, Korea Basic Science Institute, Gwangju 61186, Korea; kdpark@kbsi.re.kr

* Correspondence: chyun@jnu.ac.kr (C.-H.Y); Tel. +82-62-530-2194

Supplementary Table S1. The amino acid sequence of M16V2 and CYP102A1 mutants

Mutant	Mutated Amino Acid Residues	Reference
M16	R47L/F81I/F87V/E143G/L188Q/E267V	[1]
M16V2	R47L/F81I/F87V/E143G/L188Q/E267V/A474V/E558D/T664A/P675L/A678E/E687A/A741G/K813E/R825S/R836H/E870N/I881V/E887G/P894S/S954N/M967V/Q981R/A1008D/H1021Y/Q1022E[c]<br>Mutants A5-M697 contain underlined 20 mutated amino acids of V2 reductase domain of M16V2	[1]
A5	R47L/F81I/F87V/E143G/L188Q/E267V/I401T	[1]
B1	R47L/F81I/F87V/E143G/L188Q/E267V/D351G	[1]
C7	R47L/K59N/F81I/F87V/E143G/L188Q/G240E/E267V/S383R	[1]
D1	R47L/F81I/F87V/Y115C/E143G/I174S/L188Q/E267V/T268A/Y313F	[1]
D8	R47L/F81I/F87V/E143G/L188Q/E267V/Y334C/A335V/D369G	[1]
E10	R47L/F81I/F87V/Q110L/E143G/L188Q/N192S/Q206K/E267V/K289N	[1]
F2	R47L/Q73R/F81I/F87V/S108N/E143G/L188Q/I220M/E267V	[1]
F7	R47L/F81I/F87V/E143G/F173C/N186K/K187Q/L188Q/E267V	[1]
G1	R47L/F81I/F87V/E143G/T152A/L188Q/E267V/Q403R/V413A	[1]
M179	R47L/F81I/F87V/E143G/L188Q/N213S/E267V	[2]
M198	R47L/F81I/F87V/E143G/F158L/L188Q/E267V	[2]
M221	F11Y/R47L/F81I/F87V/E143G/L188Q/E267V/H408R	[2]
M301	R47L/F81I/F87V/S108C/E143G/T149S/L188Q/E267V	[2]
M306	R47L/F81I/F87V/M112T/E143G/L188Q/E267V/M417T	[2]
M371	D23G/R47L/F81I/F87V/F107L/D136G/E143G/L188Q/E267V	[2]
M380	R47L/F81I/F87V/L103F/D136G/E143G/N159S/L188Q/E267V	[2]
M387	F11L/R47L/F81I/F87V/Q110P/E143G/L188Q/R190Q/E267V	[2]
M524	F42L/R47L/F81I/F87V/E143G/L188Q/E267V	[2]
M601	R47L/F81I/F87V/E143G/L150F/L188Q/E267V	[2]
M620	R47L/F81I/F87V/Q109L/E143G/L188Q/D199V/E267V	[2]
M634	T1S/R47L/F81I/F87V/E143G/T146A/L188Q/E267V	[2]
M697	R47L/F81I/F87V/E143G/L188Q/E267V/K312N/D370E	[2]
M788	R47L/F81I/F87V/K113N/E143G/L188Q/E267V/N319D/L347I/S383R	[2]
M850	F11Y/R47L/D68G/F81I/F87V/E143G/L188Q/E267V/H408R	[2]

Supplementary Table S2. Detailed ^{1}H and ^{13}C assignments according to the analysis of two-dimensional spectra (COSY, HSQC and HMBC) of monacolin J

Chemical Shift(δ, ppm)

Position	^{1}H(δ), ppm	^{13}C(δ), ppm
2'a	0.90 (d, *J* = 7.0 Hz, 3H)	14.41
6'a	1.19 (d, *J* = 7.5 Hz, 3H)	23.78
*8	1.42 (m, 1H)<br>1.85 (m, 1H)	25.27
6'	2.39 (m, 1H)	29.29
2'	2.40 (m, 1H)	32.29
*7	1.55 (m, 1H)<br>1.825 (m, 1H)	34.20
*5	1.80 (m, 1H)<br>1.97 (m, 1H)	36.78
*7'	1.835 (m,1H)<br>1.90 (m,1H)	37.28
1'	1.87 (m, 1H)	37.70
*3	2.545 (m, 1H)<br>2.745(dd, *J* = 17.7, 4.7 Hz, 1H)	39.30
8'a	2.15 (m, 1H)	39.76
4	4.26 (m, 1H)	63.51
8'	4.23 (m, 1H)	65.90
6	4.71 (m, 1H)	78.50
4'	5.95 (d, *J* = 9.7 Hz, 1H)	130.23
5'	5.47(m, 1H)	130.86
4'a	-(quaternary)	133.19
3'	5.76 (dd, *J* = 9.7, 6.1 Hz, 1H)	134.02
2	-(quaternary)	173.92

Supplementary Table S3. Detailed ^{1}H and ^{13}C assignments according to the analysis of 2-dimensional spectra (COSY, HSQC and HMBC) of the major product

Chemical Shift(δ, ppm)

Position	^{1}H(δ), ppm	^{13}C(δ), ppm
2'a	0.91 (d, J = 7.0 Hz, 3H)	14.29
*8	1.91 (m, 1H)<br>1.445 (m, 1H)	25.15
2'	2.40 (m, 1H)	32.40
*7	1.55 (m, 1H)<br>1.82 (m, 1H)	34.14
*7'	1.42 (m, 1H)<br>2.04 (m, 1H)	34.93
6'	2.60 (m, 1H)	36.04
*5	1.81 (m, 1H)<br>1.975 (m, 1H)	36.77
1'	1.83 (m, 1H)	37.92
3	2.74 (dd, J = 17.7, 4.7 Hz, 1H)<br>2.55 (m, 1H)	39.30
8'a	2.18 (m, 1H)	40.33
4	4.26 (m, 1H)	63.51
8'	4.27 (m, 1H)	65.81
*6'a	3.50 (m, 2H)	67.71
6	4.72 (m, 1H)	78.53
5'	5.50 (m, 1H)	126.15
4'	5.97 (m, 1H)	130.00
3'	5.76 (dd, J = 9.7, 6.0 Hz, 1H)	134.51
4'a	-(quaternary)	136.19
2	-(quaternary)	173.92

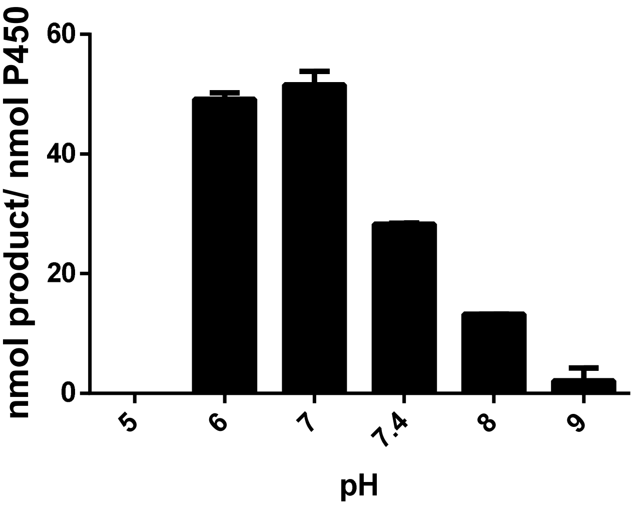

Supplementary Figure S1. pH-dependence of a hydroxylated product formation from monacolin J by CYP102A1. Effect of pH on the catalytic activity of CYP102A1 for the monacolin J hydroxylation was examined at different pH for 1 h at 37 °C. 100 mM of sodium acetate buffer and potassium phosphate buffer were used for pH 5-6, and pH 7-9, respectively.

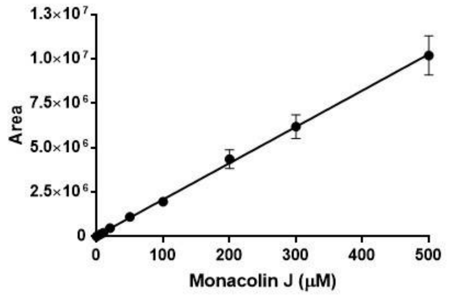

Mona-slope	20471 ± 528.4
Y-intercept when X=0.0	41798 ± 110430
R square	0.9895
Equation	Y = 20471*X + 41798

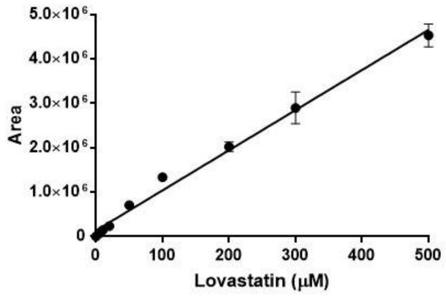

Lova-slope	9045 ± 269.3
Y-intercept when X=0.0	136883 ± 56277
R square	0.9860
Equation	Y = 9045*X + 136883

Supplementary Figure S2. Standard curves of internal standard lovastatin, and monacolin J.

Different concentrations of monacolin J and lovastatin were used at 0, 5, 10, 20, 50, 100, 200, 300, and 500 μM. The results were measured by HPLC. The values are presented as the mean ± S.E. of duplicate measurements.

References for Supporting Information

1. Kim, D.-H.; Kim, K.-H.; Kim, D.-H.; Liu, K.-H.; Jung, H.-C.; Pan, J.-G.; Yun, C.-H. Generation of Human Metabolites of 7-Ethoxycoumarin by Bacterial Cytochrome P450 BM3. *Drug Metab. Dispos.* **2008**, *36*, 2166–2170, doi:10.1124/dmd.108.021220.
2. Le, T.-K.; Jang, H.-H.; Nguyen, H.T.H.; Doan, T.T.M.; Lee, G.-Y.; Park, K.D.; Ahn, T.; Joung, Y.H.; Kang, H.-S.; Yun, C.-H. Highly Regioselective Hydroxylation of Polydatin, a Resveratrol Glucoside, for One-Step Synthesis of Astringin, a Piceatannol Glucoside, by P450 BM3. *Enzyme Microb. Technol.* **2017**, *97*, 34–42, doi:10.1016/j.enzmictec.2016.11.003.
